# Neurological Soft Signs in Individuals with Pathological Gambling

**DOI:** 10.1371/journal.pone.0060885

**Published:** 2013-04-04

**Authors:** Igor Elman, Tamara V. Gurvits, Evelyne Tschibelu, Justin D. Spring, Natasha B. Lasko, Roger K. Pitman

**Affiliations:** 1 Providence VA Medical Center, Harvard Medical School, Cambridge, Massachusetts, United States of America; 2 Cambridge Health Alliance, Harvard Medical School, Cambridge, Massachusetts, United States of America; 3 Department of Psychiatry, Massachusetts General Hospital, Harvard Medical School, Charlestown, Massachusetts, United States of America; University of Granada, Spain

## Abstract

Increased neurological soft signs (NSSs) have been found in a number of neuropsychiatric syndromes, including chemical addiction. The present study examined NSSs related to perceptual-motor and visuospatial processing in a behavioral addiction viz., pathological gambling (PG). As compared to mentally healthy individuals, pathological gamblers displayed significantly poorer ability to copy two- and three-dimensional figures, to recognize objects against a background noise, and to orient in space on a road-map test. Results indicated that PG is associated with subtle cerebral cortical abnormalities. Further prospective clinical research is needed to address the NSSs' origin and chronology (e.g., predate or follow the development of PG) as well as their response to therapeutic interventions and/or their ability to predict such a response.

## Introduction

Parallel to the ongoing expansion of legalized gambling activities is an increase in the prevalence of pathological gambling (PG) [1], [2]. Pathological gambling afflicts up to 5% of the general adult population and it costs American society an estimated $54 billion annually due to crime, decreased productivity, and bankruptcies [3]–[7]. These estimates are likely conservative, given that PG is not a conspicuous addiction, and it is devoid of typical symptoms of intoxication, needle marks, or overdose. It may only become noticeable in later stages of the illness, with the emergence of highly visible behaviors including attempted suicide in up to 24% of untreated individuals [7]–[9]. To improve prevention and treatment of PG, it is important to identify its behavioral markers and their neural correlates.

A relatively consistent finding in functional brain imaging studies of PG is failure of prefrontal cortical areas to activate when challenged by cognitive tasks that normally evoke cerebral blood flow and metabolic responses in these regions [10]–[17]. Likewise, neuropsychological impairments are commonly documented in PG patients [18]–[20], but their role in the course of the disorder remains unclear [16], as they do not reliably reflect the severity of gambling problems [21], [22]. The nonspecificity of PG neuropsychological findings may be partially attributable to the multidimensionality of the tests employed [23]. Additionally, some results may reflect poor motivation and attention [24], [25] rather than PG-related primary neuropathology, which has not yet been well defined [23].

Neurological assessment paradigms may be of value in revealing cortical abnormalities in PG. In this regard, neurological soft signs (NSSs) are reliable [26]–[28], easily administered and temporally stable [29], [30] markers of neurological compromise, which impose fewer cognitive demands than neuropsychological tests and are therefore less influenced by performance confounds [31]. In contrast to hard neurological signs localizable to a specific brain site, their soft counterparts are attributed to wider brain regions and functionally connected neuroanatomical systems, involved in integrative neurological functions such as sensory perception, coordination and motor sequencing [32], [33]. Neurological soft signs have been observed in a growing number of neuropsychiatric syndromes including mood disorders [34]–[36], obsessive-compulsive disorder (OCD) [37]–[39], post-traumatic stress disorder [26], [27], impulse control disorder [40], schizophrenia [32], [34], [41], and attention deficit hyperactivity disorder [42]. Furthermore, an inverse relationship between NSSs scores and total brain volume has been noted in psychopathological populations [27], [43] adding support to the generalized rather than localized NSSs' nature.

In a previous paper, we reported that cocaine dependence is characterized by the NSS of constructional apraxia [31]. As with PG, cocaine dependence is classified in the DSM-V draft among Substance Use and Addictive Disorders [44]. However, in addition to its representing a behavioral addiction, a substance addiction to cocaine exerts profound chemical effects on the brain that may even result in such injuries as subarachnoid/parenchymal hemorrhages [45]–[56] and infarcts [47], [50].

Because it is not confounded by exogenous neurotoxicity, PG offers a unique opportunity to test whether a purely behavioral addiction is accompanied by neurological compromise. To our knowledge, NSSs have not yet been investigated in pathological gamblers. The presence in PG of obsessive/compulsive and impulsive features each of which has been previously linked with NSSs [40], [57], [58] suggests that NSSs may also be seen in PG. Accordingly, in this project we assessed three NSSs in PG and healthy subjects. These were: a) copying two- and three-dimensional figures (as previously tested in cocaine subjects [31]); b) filtration of visual signal from noise; and c) left-right orientation in the form of reading and understanding a simple road map. These visuospatial and sensory integration tasks were selected for the present project from our comprehensive NSSs assessment battery based upon their discriminative ability in drug-dependent and other psychiatric patients [27], [31], [59] as well as their ease of administration as paper-and-pencil tasks. We hypothesized that patients with PG would be more impaired than healthy subjects on all three tasks.

## Methods

### Subjects

Twenty-one subjects who met the Diagnostic and Statistical Manual of Mental Disorders, Fourth Edition, Text Revision (DSM IV-TR) criteria for PG, and 10 non-gamblers who did not meet DSM IV-TR criteria for any disorder, were recruited by newspaper advertisement for participation in a previous study on the neurobiology of PG. The biochemical [60] and psychosocial [61] stress responsivity findings from that study have been reported elsewhere. After a full explanation of the procedures, all subjects gave written informed consent to the McLean Hospital Institutional Review Board-approved protocol. Those with any cognitive impairment that precluded informed consent based on clinical interview and the assessments instruments (see below) were excluded from study participation. Subjects were diagnosed by a research psychiatrist using a best estimate format utilizing all available sources of information including clinical history, interview, and the following psychodiagnostic instruments: the Structured Clinical Interview for DSM-IV (SCID [62]); the South Oaks Gambling Screen (SOGS [63]); the DSM PG checklist (DSMIV-TR [64], [65]); and the Addiction Severity Index [66]. All subjects were right handed as determined by the Edinburgh Handedness Inventory [67], and scored at least 28 on the Mini Mental Status Examination (MMSE [68]). Furthermore, they were in good physical health as ascertained by the Cornell Medical Index Health Questionnaire [69].

The exclusion criteria included left handedness, lifetime history of dementia, schizophrenia, or other psychotic disorder, bipolar disorder, anxiety disorder, current drug or alcohol dependence, past but not current PG, or major depression with onset prior to PG. We also excluded potentially confounding neurological conditions, such as seizure disorder, head trauma accompanied by loss of consciousness greater than 10 minutes, brain surgery, multiple sclerosis, and Parkinson's disease, as well as potentially confounding medical conditions such as chronic obstructive pulmonary disease, coronary artery disease, diabetes, obesity (body mass index ≥30), congestive heart failure, hypertension, renal diseases, cirrhosis, HIV-positive status and AIDS. Recent drug and alcohol consumption was ruled out by negative results on urine toxicology screen and breathalyzer.

### Procedures

The three tasks were administered over one session in the following order: Copy Figure Test (CFT), Detection and Recognition of an Object Test (DROT) and Road Map Test (RMT). None of the tasks was timed. Responses on the DROT and the RMT were recorded by a research assistant seated next to the subject. Both the subject and the examiner were ‘blind’ to the study's hypothesis.

**Figure 1:**
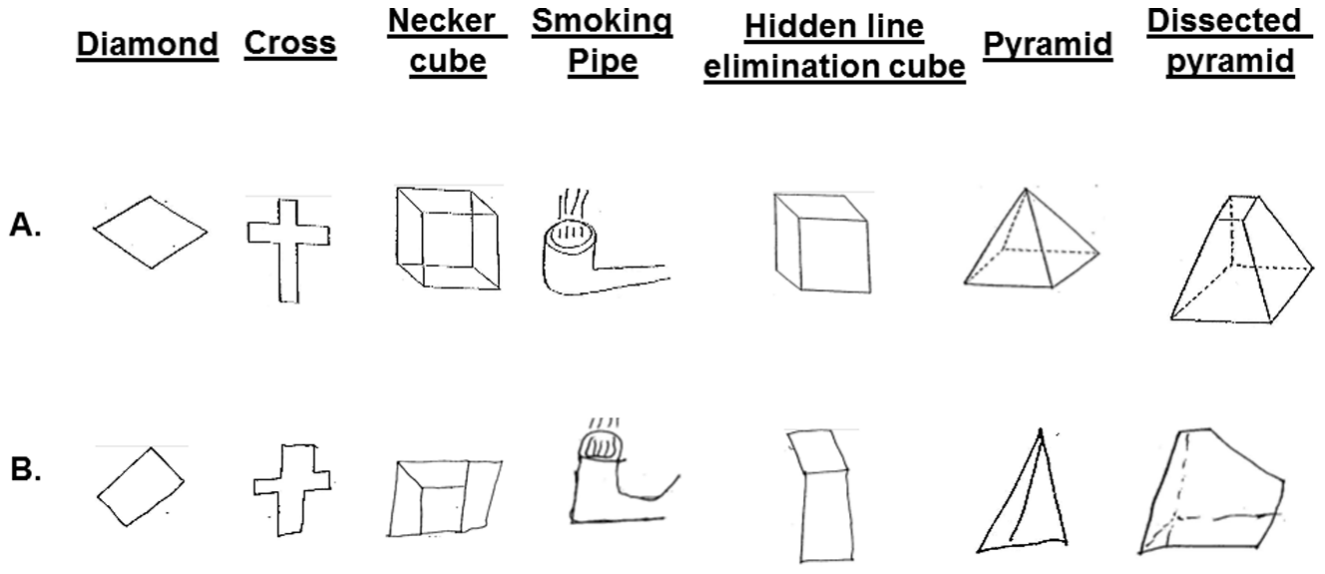
The two-dimensional (diamond and cross) and three-dimensional (Necker cube, smoking pipe, hidden line elimination cube, pyramid and dissected pyramid) figures copied by the subjects (Panel A). Examples of PG subjects' performance on the Copy Figure Test (Panel B).

The CFT [70], [71] is perceptual-motor in nature and comprises two-dimensional (diamond and cross) and three-dimensional (Necker cube, smoking pipe, hidden line elimination cube, pyramid and dissected pyramid) figures (Figure 1A). Subjects were instructed to copy each figure exactly as it appeared to them with a pen. They were allowed to look at each figure as often as needed. They were further instructed neither to erase any lines nor to draw any lines that did not appear in the figure they copied.

**Figure 2:**
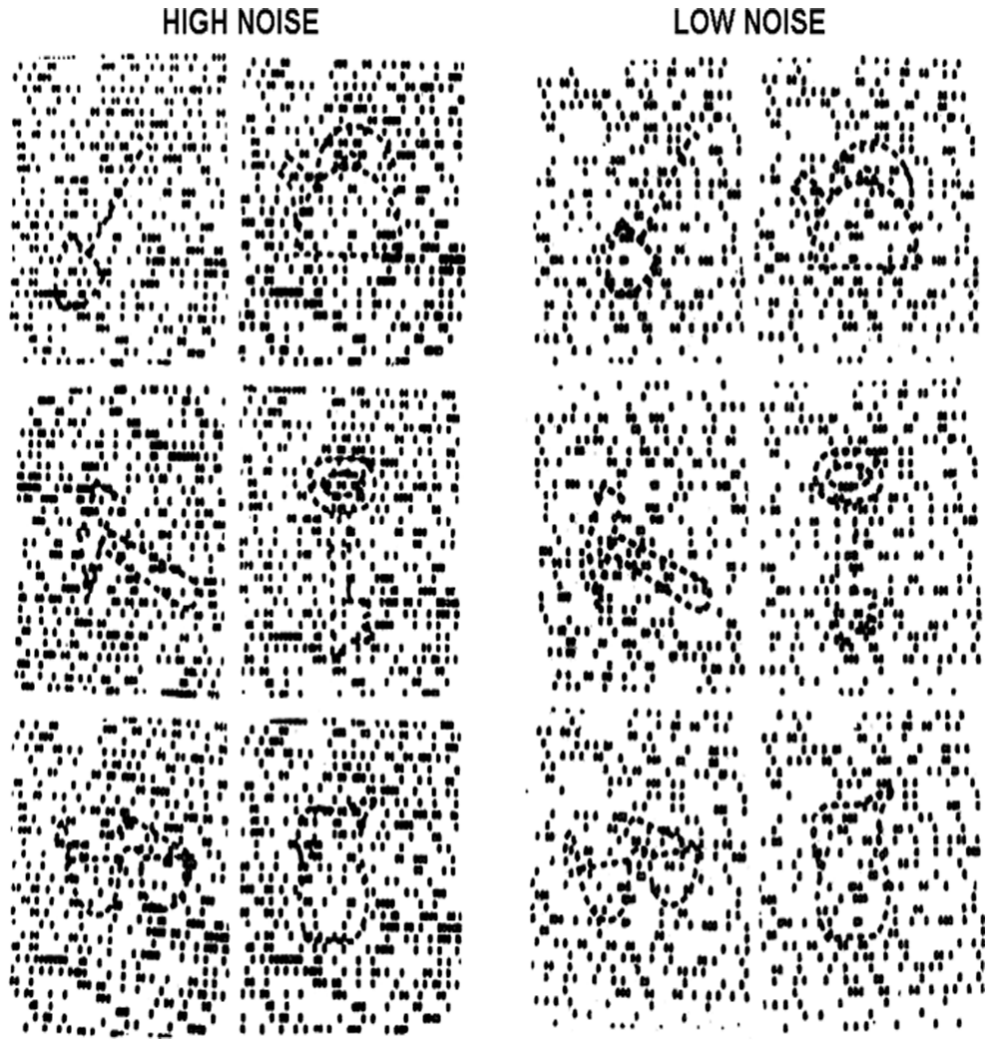
Detection and Recognition of an Object Test (DROT). “High noise” and “low noise” sets were presented separately, with the latter following the former. Subjects were instructed to identify the object embedded in the noise.

The DROT [72], [73] consisted of two sets of the same six images (Figure 2). Each image depicted a single basic household object, namely key, shovel, pitcher, eyeglasses, hammer, and kettle. However, the object recognition was complicated by background “noise,” consisting of a field of black squares of two different densities, namely 35 and 15 squares per line. Subjects viewed all six objects with the denser (more difficult) background first, followed by all six objects with the less dense (less difficult) background. They were instructed to identify all objects and were allowed to bring the page as close to the eyes as desired.

**Figure 3:**
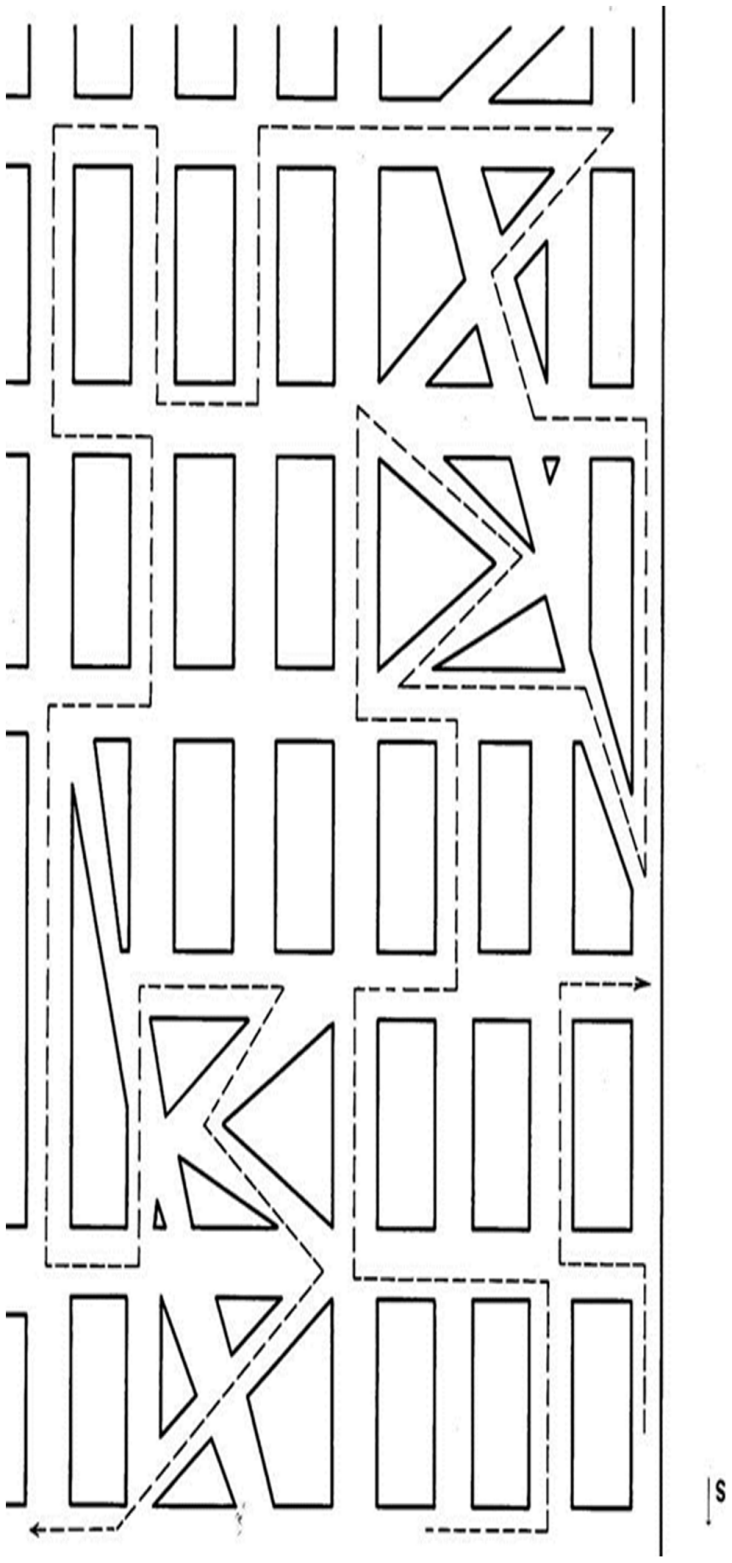
The Money Road Map Test (RMT). The continuous dotted line represents the path followed by the researcher's pen. Subjects were asked at each successive turn to indicate whether it was right or left. The smaller dotted line in the lower right serves as a practice trial.

The RMT [74] is designed to evaluate directional sense in visuospatial processing (Figure 3). Subjects were presented with a map of an imaginary town, with a delineated route containing 32 intersections. They were instructed to imagine driving this route and to indicate at each intersection whether the route turned left or right. The research assistant followed the route with a pencil and marked R or L in accordance with the verbal response at each intersection. The map remained in a fixed position in front of the subject, and they were not allowed to move it. Each subject's familiarity with the task was confirmed via a brief practice trial.

The CFT was scored by a dually trained psychiatrist and neurologist, who not only was blind to diagnosis but had never seen the subjects, utilizing a four-point scoring convention for each figure. Zero (0) coded perfect or near perfect reproduction; 1 coded mild distortion or rotation; 2 coded moderate distortion or rotation, or severe micropsy or a loss of three-dimensionality; and 3 coded gross distortion of the basic gestalt or a virtually unrecognizable image. On the DROT, number of failed identifications was scored. On the RMT, number of wrong turns was scored.

Demographic variables were analyzed by Student's t-tests or Fisher's exact tests as appropriate. Because most of the CFT, DROT, and RMT data were ordinal and not normally distributed, they were summarized as both median and mean ± standard deviation (SD). The univariate nonparametric Wilcoxon rank-sum test was used to compare groups. Significance was defined as *p*<0.05, one-tailed, with more abnormalities predicted in the PG group.

## Results

Table 1 presents demographic and psychometric data for the two groups. These data demonstrate that pathological gamblers were not significantly different from healthy controls with respect to age, race, gender, years of education, performance on the MMSE, and consumption of alcohol. As planned, there were conspicuous differences in SOGS score and the number of DSM-IV TR PG criteria met.

**Table 1:** Demographic and Clinical Characteristics (Means ± SDs or Ratios) of Study Participants.

Variable	PG (n = 21)	Control (n = 10)	T-test (df = 29)	
			t	*p*
Age (year)	45.5±9.9	43.6±14.2	0.44	0.66
Education (year)	15.0±2.8	15.1±1.4	−0.16	0.88
MMSE (score)	29.3±0.9	29.4±1.1	−0.19	0.86
Alcohol (drink/week)	1.0±3.0	0.6±1.3	0.44	0.66
DSM-IV-TR PG criteria met	7.3±1.2	0.0±0.0		
SOGS	13.5±3.8	0.0±0.0		
			Fisher's exact test	
Gender (M/F)	13/8	5/5		0.74
Race (W/B)	10/11	7/3		0.72

Figure 1B presents examples of mistakes made by PG subjects on the CFT. Table 2 presents the group medians and means **±** SDs for each CFT figure separately and for the average score of all 7 figures, as well as the DROT and RMT score means and medians, and the results of the group comparisons. With the exception of the smoking pipe figure and the pyramid figure (for which there was a trend), all tests revealed significantly poorer performance in the PG group. Performance on the hidden line elimination- and Necker cubes was dramatically poorer in the PG subjects. Notably, the latter test is characterized by ambiguous front-back orientation necessitating visuospatial ability to shift attention between two equally plausible figural spatial representations [75].

**Table 2:** Group medians and mean (±SDs) for the performance indices on the Copy Figure, Detection and Recognition of an Object and the Road Map tests.

Task	PG (n = 21)	Control (n = 10)	Wilcoxon Exact Test
	Median Mean ± SD	Median Mean ± SD	*p*
CFT (score; 0–3)			
1. Diamond	1 0.6±0.5	0 0.2±0.4	0.03
2. Cross	1 1.0±0.7	0 0.4±0.5	0.01
3. Necker cube	3 2.1±1.1	0 0.6±0.8	<0.001
4. Smoking pipe	0 0.6±0.9	0 0.2±0.4	0.13
5. Hidden elimination cube	2 2.0±1.0	0.5 0.5±0.5	<0.001
6. Pyramid	1 1.2±1.1	0.5 0.5±0.5	0.06
7. Dissected pyramid	1 1.8±1.0	0 0.6±1.0	<0.001
Average	1.4 1.3±0.7	0.3 0.4±0.4	<0.001
DROT error (#)			
High noise	4 3.8±1.2	3 2.8±0.9	0.01
Low noise	3 2.8±1.3	1 1.4±1.3	0.01
RMT error (#)	4 5.1±5.1	1 1.0±1.2	0.02

Repeating the analyses after excluding ten smokers (all in the PG group; among them are two subjects with respective cocaine and alcohol dependence, both in full sustained remission), the group effect remained significant for the CFT average score (p = 0.002), for the high (p = 0.03) and low (p = 0.0005) noise DROT errors and for the RMT errors (p = 0.03).

## Discussion

In this study we identified several signs in pathological gamblers reflecting their diminished ability to recognize and construct objects and orient them in space. These dysfunctions have not yet been addressed in literature on neuropsychological disturbances in PG. In comparison to healthy subjects, pathological gamblers showed substantially worse performance on copying two- and three-dimensional figures, recognizing objects against background noise, and discriminating left from right turns on a map. Methodological similarities between the present study and our prior study of cocaine dependence [31] included enrollment of subjects with addictive disorders and use of a standard copy figure task. There were differences in the type of addiction and in the number of tasks performed by subjects. Overall these results provide further support for subtle neurobiological impairment in a behavioral addiction that is not confounded by exogenous chemical use. Our data are also consistent with a substantial body of literature documenting neuropsychological impairments in PG patients [18]–[20], and they extend prior findings by suggesting that the impairments are not restricted to the cognitive domains addressed by neuropsychological testing but also generalize to the sensorimotor domain.

Several brain regions influence the drawing of three-dimensional figures, but as evident from research on cortically damaged patients [76] and from neuroimaging work [75], [77] the most important of the regions is the parietal cortex. Ventral striatum and related mesolimbic dopaminergic circuitry are traditionally considered to be a key component of reward system involved in addiction [78], and it is commonly hypothesized that changes in the mesolimbic pathways underlying motivational processes are responsible for transforming regular drives into heightened incentive salience assigned to addiction-related cues [79]. However, recent research suggests a novel factor in the mechanisms underlying incentive sensitization by implicating parietal cortex in the control exerted over striatal signals of salience via integration of visuospatial, motor and cognitive (e.g., hedonic value and categorical boundaries) inputs [80]. In addition to these theoretical considerations, an abundant clinical literature demonstrates parietal cortex changes in the context of chronic addictive behaviors [81], [82]. Hence NSSs examination may support the need to focus on this important region and on its role in the pathophysiology of PG.

A limitation of the cross-sectional design employed here is its inability to resolve the origin of elevated NSSs in PG. One possibility is that they are preexisting vulnerability markers [83]. A growing body of work points to compromised cortical function reflected in NSSs that precedes the emergence of mood, anxiety [57], psychotic [84]–[86] and obsessive-compulsive [57], [87] symptoms. Neurological soft signs are also commonly observed in mentally healthy relatives of schizophrenic patients [88]–[91], further suggesting their preexisting and inheritable trait-like nature. Notably, as suggested by twin studies, PG has a robust genetic component ranging from 50 to 60% [92]. Greater premorbid hyperactivity, impulsivity, and antisociality have been found in PG subjects [93].

A second possible origin of NSSs in PG is that they are acquired, e.g., they are a consequence of excessive gambling. People who gamble lose money, and a consequence of losing money may be increased stress, possibly leading to brain alterations. Pathological gambling is indeed associated with an exaggerated sympathoadrenal tone suggestive of heightened levels of stress and arousal [94] at baseline [95], [96] and while engaged in gambling [8], [9], [97]–[100]. Subjects with PG have greater amygdala activation in response to the alpha-2 adrenergic antagonist, yohimbine [60]. Research in laboratory animals [101] and humans [102], [103] has shown that increased sympathetic activity may cause vasospasm and microthrombosis resulting in diminished cerebral perfusion. It would be of interest to test whether antiadrenergic agents (e.g., clonidine or prazosin) might moderate the NSSs observed here. However, the reversibility of NSSs is questionable [39], given that this has only been found in some [104] but not in all OCD patients [105]–[107], and not in patients with bipolar disorder [108] or schizophrenia [107], [109]. In sum, resolution of the risk factor vs. acquired origin interpretation of the observed NSSs in PG, as well as NSSs' possible response to treatment and/or their ability to predict [37], [110] such a response (as has been shown for OCD patients) will require prospective clinical trials.

The present design is unable to inform the question as to whether the same visual agnosia displayed by the PG subjects on the DROT is not likewise implicated in their constructional apraxia on the figure copying task. Disentangling this would require an exclusively motor processing task that does not involve visual input [27]. Such tasks are included in the full assessment battery of previously reported NSSs [27], which assesses motor coordination and both motor and sensory integration.

In conclusion, the data presented here shed light on the neurological function of patients with PG and suggest that NSS examination has heuristic value for illuminating brain abnormalities in this disorder. Pathological gambling offers a unique model as it represents an addictive behavior in the absence of the potentially confounding pharmacologically neurotoxic effects of chemical substances. Therefore these findings provide a new perspective in the exploration of addiction neurobiology.
